# Maternal Risk Factors and Their Effect on Outcome and Procedure-Related Complications in Cordocentesis: A Multicenter Retrospective Study

**DOI:** 10.3390/jcm12216841

**Published:** 2023-10-30

**Authors:** Philipp Kosian, Karl-Philipp Gloning, Ute Germer, Brigitte Strizek, Christoph Berg, Ulrich Gembruch, Annegret Geipel

**Affiliations:** 1 Department of Obstetrics and Prenatal Medicine, University Hospital Bonn, 53127 Bonn, Germanyannegret.geipel@ukbonn.de (A.G.); 2 Pränatal-Medizin München, 80639 Munich, Germany; 3 Department of Prenatal Medicine, St. Josef Hospital, 93053 Regensburg, Germany; 4 Division of Prenatal Medicine and Gynecologic Sonography, Department of Obstetrics and Gynecology, University of Cologne, 50931 Cologne, Germany

**Keywords:** fetal blood sampling, maternal risk factors, procedure-related complications, fetal loss

## Abstract

Background: Cordocentesis is used in clinical situations in which lower-risk diagnostic procedures do not deliver the desired results. The aim of this study was to evaluate the risk for procedure-related complications and fetal loss in correlation to maternal risk factors. Methods: This is a multicenter retrospective study investigating the complications, risk factors and perinatal outcome of diagnostic cordocentesis between 1998 and 2019 in three different centers. Results: A total of 1806 cordocenteses were performed and procedure-related complications (IUFD within 48 h, contractions, bradycardia, unsuccessful puncture, chorioamniotic separation) were noted in 1.6% of cases. Fetuses with chromosomal aberrations, intrauterine growth restriction and hydropic fetuses had a significantly higher rate of fetal loss compared to other indications. Fetal blood sampling (FBS) performed before 17^+0^ weeks of gestation was associated with a higher risk of procedure-related complications. Maternal BMI ≥ 40 increased the risk for fetal loss, whereas maternal age, number of previous miscarriages, number of previous abortions, history of vaginal bleeding or nicotine abuse did not affect the risk for complications or overall fetal loss rate. Conclusions: In the hands of experienced operators, FBS is a safe way to further fetal diagnostics, and the risk of complications is low.

## 1. Introduction

Fetal blood sampling (FBS) has broadened our ability to diagnose, monitor and address a wide array of fetal health conditions, making it a cornerstone of contemporary fetal medicine. Prenatal medicine has witnessed remarkable progress over the years, with fetal blood sampling standing as a testament to the extraordinary advances in our understanding of fetal health and development. It is a procedure that allows healthcare providers to access a wealth of invaluable information about the fetus, its genetic makeup, blood composition and overall well-being. Whether it is for the early detection of genetic disorders, monitoring of fetal anemia or the diagnosis of infections, this procedure provides insights that shape the course of prenatal care and decision making for both the expectant mother and the medical team [1]. Apart from the diagnostic aspect, FBS also allows for fetal therapy, including intrauterine blood transfusions in cases of fetal anemia or antiarrhythmic therapy in cases of refractory fetal arrhythmia after unsuccessful transplacental treatment [2]. Laboratory markers like white blood cell and differential counts, red cell indices, blood gas analysis, microproteins and thyroid hormone levels can be analyzed. However, it is important to compare these values with reference values appropriate for gestational age, as these might diverge significantly from newborn levels [1].

Since amniocentesis (AC) or chorionic villi sampling (CVS) are the commonly used invasive diagnostic procedures that provide the desired information in most cases, FBS is reserved for clinical situations in which the aforementioned lower-risk diagnostic procedures do not deliver or only delay the delivery of the desired results. Since AC and CVS can be used for karyotyping, the confirmation of suspected fetal anemia and nonimmune fetal hydrops with suspected fetal anemia have been the most common indication for FBS in recent years [1]. Sampling site is selected upon placenta localization, fetal position and operator experience. The most common way of access is the placental insertion of the umbilical vein, as fetal movement will at least affect the procedure. When cordocentesis has failed, FBS of the fetal intrahepatic vein (IHV) is possible. Certain advantages such as decreased fetal blood loss and fetomaternal hemorrhage have been described [3]. Cardiac access is rarely performed due to its high fetal loss rate [1,4]. An anterior wall placenta facilitates the FBS, but the risk for fetomaternal hemorrhage or fetal loss is increased [5], whereas in patients with posterior wall placenta and polyhydramnios, the fetus might hinder access. Complications from cordocentesis include fetal bleeding from the puncture site in 20–32% of cases, which is associated with fetal loss [1,6], and cord hematoma. Fetomaternal hemorrhage occurs in up to 40% of cases and is significantly more common in anterior wall placenta, in procedures longer than three minutes and in cases of two or more needle insertions [7]. Further complications are bradycardia (5–10%) [1,6], infection (1%), failure to obtain fetal blood (5–10%) [3] and fetal loss (0.6–1.9%) [6,8].

To our knowledge, there are no data on maternal risk factors influencing procedure-related complications or fetal loss in cordocentesis. The aim of this study was, therefore, to evaluate if maternal risk factors, the indication, technical aspects or gestational week have an impact on procedure-related complications and fetal loss.

## 2. Materials and Methods

This is a multicenter retrospective study investigating procedure-related complications, fetal loss, maternal risk factors and perinatal outcome in diagnostic cordocentesis between 1998 and 2019 in three different centers (Department of Obstetrics and Prenatal Medicine, University Hospital Bonn; Pränatal-Medizin München, Munich; and Department of Prenatal Medicine, St. Josef Hospital, Regensburg). All 1806 procedures were performed by highly trained and educated specialists in fetal medicine. The technique of the cordocentesis was the same in all three centers and included the following steps: Before the procedure, the mother and fetus were monitored through ultrasound to identify the position of the fetus and the umbilical cord. The expectant mother was informed about the procedure and its potential risks and benefits. Informed consent was obtained before proceeding. The mother’s abdominal area was sterilized, followed by needle insertion under ultrasound guidance. Once the needle was correctly positioned in the umbilical cord, a small sample of fetal blood was withdrawn into a syringe. After the blood sample was collected, the needle was withdrawn, and follow-up monitoring with ultrasound was performed to ensure that there were no immediate complications.

Ethical review and approval were waived in view of the retrospective nature of the study. The Viewpoint database (Version 5.6.28.60.) was searched for eligible patients. Then, indication, technical aspects, fetal loss rate, complications within 48 h (intrauterine fetal death (IUFD), contractions, bradycardia, chorioamniotic separation, unsuccessful puncture) and perinatal outcome were evaluated and correlated with maternal risk factors (maternal age, BMI, prior miscarriages, prior abortions, history of vaginal bleeding prior to FBS and nicotine abuse). To analyze if early FBS was associated with a higher rate of complications, we compared cordocentesis performed before and after 20 weeks of gestation and before and after 17 weeks of gestation. Follow up data and outcome were extracted from the Viewpoint database and perinatal records. In total, 10.8% of cases were lost to follow up. Data analysis was performed with IBM SPSS software version 27 (SPSS Inc., Chicago, IL, USA). Differences between subgroups were calculated with the Pearson Chi Quadrat and Mann–Whitney U test wherever appropriate. Multiple binary regression analysis was performed to analyze the impact of maternal risk factors, gestational age and technical aspects on procedure-related complications and total fetal loss rate. A *p*-value < 0.05 was considered significant.

## 3. Results

In total, 1806 FBS (1760 singletons, 46 twins) with cordocentesis were performed between 1998 and 2019. The most common indications were fetal malformation or suspected chromosomal aberration (75.1%), suspected chromosomal aberration from prior genetic testing (3.9%), suspected fetal infection (3.3%), IUGR (intrauterine growth restriction) (3.3%), hydrops fetalis (3.2%) and suspected fetal anemia (3.0%) (Table 1). In 1.6% of cases (29/1806), complications within 48 h were observed (Table 1 and Table 2). Within 48 h, there were eleven fetal demises (details in Table 3), and the total fetal loss rate excluding cases of termination of pregnancy was 4.8% (*n* = 87). Of the fetal losses, 23/87 (26.4%) had an abnormal karyotype, 50/87 (57.5%) had malformations or a syndromal disorder and 13/87 (14.9%) were hydropic. There were four late aborts occurring between 3 and 27 days after intervention. In 2.5% (*n* = 46) of cases, cordocentesis was performed in twins, resulting in IUFD in one case with twin anemia polycythemia sequence (TAPS) and unsuccessful FBS. Compared to singleton pregnancies, we did not observe a higher rate of complications (*p* = 0.16) or fetal loss (*p* = 0.52) (Table 4).

In 38 (2.1%) pregnancies, repeated cordocentesis was necessary, which did not increase complications. In total, 95 punctures (5.3%) were performed before 20^+0^ weeks of gestation with a higher complication rate, but this did not reach statistical significance (<20^+0^ weeks of gestation 3.2%, *n* = 3/95; ≥20^+0^ weeks of gestation 1.5%, *n* = 25/1710; *p* = 0.18). In our binary multiple regression analysis, we observed a significantly higher risk of procedure-related complications in FBS performed <17^+0^ weeks of gestation (20%, *n* = 2/10) compared to ≥17^+0^ weeks of gestation 1.4%, *n* = 26/1796; *p* = 0.02) and in hydropic fetuses (8.8%, *n* = 5/52) compared to non-hydropic fetuses (1.3%, *n* = 23/1748; *p* = 0.006), whereas the way of access (*p* = 0.60) or sampling site (*p* = 0.94) did not affect the rate of complications.

In our binary multiple regression analysis, fetuses with chromosomal aberrations (7.2%, *n* = 23/320) compared to fetuses with a normal karyotype (4.6%, *n* = 59/1277; *p* = 0.03), fetal growth restriction (8.6%, *n* = 6/70) compared to other indications (4.7%, *n* = 81/1733; *p* = 0.04) and hydropic fetuses (22.8%, *n* = 13/57) compared to non-hydropic fetuses (4.2%, *n* = 74/1745; *p* = 0.001) had a significantly higher rate of fetal loss.

Maternal age and overall maternal BMI did not increase the risk for procedure-related complications or fetal loss, but we did observe a higher fetal loss rate in class III obesity (30.8%, *n* = 4/13) compared to patients with a BMI < 40 (4.7%, *n* = 83/1785; *p* = 0.001). In one of those cases, the fetus was hydropic. Number of previous miscarriages, number of previous abortions, history of vaginal bleeding and nicotine abuse did not increase complications, nor were these factors associated with fetal loss (Table 5 and Table 6).

The median gestational week at birth among live births was 37^+3^ (IQR 7), and the median birthweight was at the 30th percentile (IQR 48). There was a live birth rate of 45%, and termination of pregnancy was performed in 34.1% of cases. We observed a higher rate of preterm delivery in pregnancies with hydropic fetuses (median week of gestation at birth was 32; IQR 8) compared to non-hydropic fetuses (median week of gestation at birth was 37; IQR 7; *p* = 0.04).

## 4. Discussion

This is the first study investigating maternal risk factors and their effect on procedure-related complications and fetal loss in fetal blood sampling. We have demonstrated that obesity class III affects the overall fetal loss rate, whereas other investigated maternal risk factors did not affect the outcome. More data on maternal risk factors affecting procedure-related risks or fetal loss are available for amniocentesis (AC) and chorionic villus sampling (CVS).

Procedure-related loss for AC is reported between 0.13% and 0.27%, and for CVS at around 0.22%. Fetal loss rates for both diagnostic procedures have decreased over time in high-volume centers with experienced operators [9,10,11]. Vaginal bleeding prior to the procedure (5.9% versus 3.8%), three or more abortions in the first trimester or one or more abortions in the second trimester (6.9% versus 3.5% in controls without a history of abortion) increased the risk of pregnancy loss after amniocentesis [12]. In our case series, patients had a maximum of two abortions in their medical history, which was not associated with an increase in adverse outcomes. Likewise, prior vaginal bleeding was not associated with an increased risk for fetal loss (*p* = 0.93) or procedure-related complications (*p* = 0.51) in our study.

Harper et al. investigated the effect of maternal BMI on the total fetal loss rate in AC and CVS and found no overall difference in obese (*n* = 2742) vs. nonobese (*n* = 8037) women. If, however, BMI < 25 was compared to BMI ≥ 40 (class III obesity), a significantly higher fetal loss rate was seen in the latter group [13]. This is in accordance with our observation of an increased risk for overall fetal loss in patients with class III obesity (4/9, 44.4%; *p* = 0.001). Recent published data from the Danish National Birth Cohort demonstrated that obesity is independently associated with a higher rate for stillbirth and neonatal death [14], suggesting that this might not be entirely caused by FBS.

Papantoniou et al. demonstrated in second trimester amniocentesis a significantly higher fetal loss rate in women aged > 40 (5.1%) compared to those 20–34 years of age (2.5%) [15]. In contrast to the data in this study, we found no correlation between maternal age and an increased fetal loss rate.

In the literature, complication rates for FBS range between 1.4% and 6.9% [6,8,16]. The comparability of individual studies is particularly hampered by the heterogeneous study populations. Tanvisut et al. recently reported cordocentesis-associated fetal loss and risk factors in 6650 singleton pregnancies between 16 and 24 weeks of gestation. The most common indication, representing 75% of the study population, was the risk for severe fetal thalassemia. Twin pregnancies, pregnancies with fetal malformation or chromosomal abnormality and patients with an underlying maternal medical disease were excluded. Procedure-related complications in this study were defined as fetal bradycardia, bleeding from puncture site, duration of bleeding and total fetal loss. Prolonged bleeding from puncture site and fetal bradycardia were independent risk factors for fetal loss. The overall fetal loss rate was reported as 1.6% compared to 1% in the control group. This is lower compared to our study (4.8%), which is explained by the exclusion of pregnancies with fetal structural or chromosomal abnormalities in the study by Tanvisut et al. [6].

Liao et al. investigated 2010 procedures between 1991 and 2004 and included pregnancies with risk for severe thalassemia (59% of patients) and the need for rapid karyotyping in 30% of cases. In 2.6% of cases, fetal malformation was observed; in 9.6% of cases, fetal hydrops was observed. Procedure-related complications were transient bleeding (19.8%) and fetal bradycardia (4.9%). The total fetal loss rates before or after 24 weeks of gestation were 1.3% and 0.6%, respectively, and differed significantly [17]. In our study, collective risk for procedure-related complications increased in cordocentesis performed before 17 + 0 weeks of gestation. We performed FBS between 15 + 6 and 40 + 0 weeks of gestation, and the indication in most of our cases was fetal malformation or suspected chromosomal aberration. Finally, in 17.9% of fetuses, chromosomal aberrations were confirmed, which contributes to the high rate of total fetal loss (4.8%) and termination of pregnancy (34.1%).

Bernaschek et al. analyzed 166 FBS and also reported a high rate of complications (55.6%) in cases with hydrops such as chorioamnionitis, abort, IUFD or preterm delivery; these were also significantly higher compared to other indications [18].

In accordance with the literature, we did not observe more complications in twin compared to singleton pregnancies. Tongprasert et al. investigated 59 cordocentesis in 30 multifetal pregnancies and did not detect fetal loss within two weeks after the procedure. The total fetal loss rate was at 9.5%, and in 22% of cases, transient bradycardia was reported [19]. In our series of 46 FBS in twin pregnancies, we did not note transient bradycardia but one IUFD within 48 h after an unsuccessful puncture in a pregnancy complicated by TAPS. Boupaijit et al. described a higher rate of complications (3.6% vs. 1.3%) if the placenta was penetrated [5], which we could not confirm in our study population.

Limitations of our study include the retrospective design, the missing control group and the lack of outcome in 10.8% of cases. Furthermore, maternal diseases and their possible impact on complications and fetal loss could not be analyzed.

## 5. Conclusions

We analyzed a total of 1806 cordocentesis and found an increased risk for fetal loss in fetuses with chromosomal aberrations, fetal growth restriction and hydropic fetuses. Cordocentesis performed before 17^+0^ weeks of gestation was associated with a higher risk of procedure-related complications. Maternal BMI ≥ 40 increased the risk for fetal loss, whereas other maternal risk factors did not influence the outcome. To summarize our data, when performed by experienced operators, FBS is a safe way to further fetal diagnostics, and the risk of complications is low.

## Figures and Tables

**Table 1:** Fetal characteristics and procedure details in 1806 cordocentesis. Variables: indication, puncture site, transplacental puncture, procedure-related complications within 48 h. Abbreviations: IUGR: intrauterine growth restriction; IUFD: intrauterine fetal death.

Variables		*n* (%)
Indication	Fetal malformation/suspected syndrome	1357 (75.1%)
	Suspected chromosomal aberration from prior genetic testing	71 (3.9%)
	Suspected infection	59 (3.3%)
	IUGR	60 (3.3%)
	Hydrops fetalis	57 (3.2%)
	Suspected anemia	55 (3.0%)
	Fetal intracranial hemorrhage	24 (1.3%)
	Other	123 (6.9%)
Puncture Site	Placental cord insertion	1388 (76.9%)
	Free loop	274 (15.2%)
	Fetal cord insertion	28 (1.5%)
	Missing information	116 (6.4%)
Transplacental puncture		941 (52.1%)
Procedure-related complications within 48 h	Overall	29 (1.6%)
	IUFD	11 (0.6%)
	Contractions	7 (0.4%)
	Bradycardia	5 (0.3%)
	Unsuccessful	4 (0.2%)
	Chorioamniotic separation	2 (0.1%)

**Table 2:** Indications for FBS with noted complications within 48 h. IUGR: intrauterine growth restriction; NST: nonstress test; IUFD: intrauterine fetal death.

Indication	IUFD within 48 h (*n*)	Contractions (*n*)	Bradycardia (*n*)	Unsuccessful (*n*)	Chorioamniotic Separation (*n*)	Number of Complications (*n*)
Fetal malformation/suspected syndrome	4	5	4	3	1	17
Suspected chromosomal aberration from prior genetic testing	0	1	0	0	0	1
Suspected infection	0	1	0	0	0	1
IUGR	1	0	0	0	0	1
Hydrops fetalis	3	0	1	0	1	5
Suspected anemia	1	0	0	1	0	2
Fetal intracranial hemorrhage	1	0	0	0	0	1
Abnormal NST	1	0	0	0	0	1
Total number	11	7	5	4	2	29

**Table 3:** Characteristics of cases associated with fetal death within 48 h after cordocentesis. GA: gestational age; NST: nonstress test; IUFD: intrauterine fetal death; IUGR: intrauterine growth restriction; TAPS: twin anemia polycythemia sequence.

	Indication	GA at FBS (wks)	Karyotype
IUFD within 24 h			
1	Hydrops	17 + 1	not done
2	Fetal malformation	32 + 4	47, XY, +18
3	Hydrops	27 + 2	normal
4	Sinus venous thrombosis and subdural hematoma	20 + 0	normal
5	Hydrops	28 + 2	normal
6	Fetal malformation	27 + 6	47, XY, +21
7	IUGR	27 + 4	normal
IUFD within 48 h			
1	Abnormal NST	28 + 4	not done
2	Anemia (TAPS)	21 + 4	not done
3	Fetal malformation	18 + 1	not documented
4	Fetal malformation	20 + 4	normal

**Table 4:** Outcome in fetuses after cordocentesis. IUFD: intrauterine fetal death.

Outcome	*n* (%)
Live birth	812 (45.0%)
Termination of pregnancy	615 (34.1%)
Late abort	4 (0.2%)
IUFD within 24 h	7 (0.4%)
IUFD within 48 h	4 (0.2%)
IUFD after 48 h	72 (4.0%)
Total fetal loss rate	87 (4.8%)
Death within 1 week after birth	52 (2.9%)
Death after 1 week of birth	43 (2.4%)
Missing information	196 (10.8%)

**Table 5:** Multiple binary regression analysis: impact of maternal risk factors, fetal characteristics and technical aspects on procedure-related complications. A *p*-value < 0.05 was considered significant. Variables: FBS (fetal blood sampling); access way; sampling site, fetal malformation, hydrops fetalis, IUGR (intrauterine growth restriction), maternal age, BMI (Body Mass Index), number of miscarriages, number of abortions, nicotine abuse, prior vaginal bleeding.

Variables	Standard Error	OR (95% Confidence Interval)	*p*-Value
FBS < 17 + 0 wks	1.205	17.815 (1.68–188.98)	0.017
Access way	0.392	1.228 (0.57–2.65)	0.601
Sampling site	0.355	1.028 (0.512–2.06)	0.939
Fetal malformation	0.687	1.026 (0.27–3.94)	0.970
Hydrops fetalis	0.207	1.775 (1.18–2.67)	0.006
IUGR	1.194	1.482 (0.14–15.4)	0.742
Maternal age	0.041	0.965 (0.89–1.05)	0.386
BMI	0.053	0.997 (0.90–1.11)	0.958
Number of miscarriages	0.373	1.107 (0.53–2.30)	0.786
Number of abortions	0.387	1.265 (0.59–2.70)	0.545
Nicotine abuse	1.048	0.616 (0.08–4.80)	0.644
Prior vaginal bleeding	1.067	2.027 (0.25–16.40)	0.508

**Table 6:** Multiple binary regression analysis: impact of maternal risk factors, fetal characteristics and technical aspects on total fetal loss rate. A p-value < 0.05 was considered significant. Variables: gestational age (GA), access way, sampling site, karyotype, fetal malformation, hydrops fetalis, IUGR (intrauterine growth restriction), maternal age, Body Mass Index (BMI) ≥ 40, number of miscarriages, number of abortions, nicotine abuse, prior vaginal bleeding.

Variables	Standard Error	OR (95% Confidence Interval)	*p*-Value
GA	0.026	1.021 (0.97–1.07)	0.426
Access way	0.212	0.874 (0.58–1.32)	0.526
Sampling site	0.214	0.937 (0.62–1.43)	0.761
Karyotype	0.269	1.798 (1.06–3.04)	0.029
Fetal malformation	0.442	1.574 (0.66–3.74)	0.305
Hydrops fetalis	0.138	1.822 (1.39–2.39)	0.001
IUGR	0.604	3.477 (1.07–11.35)	0.039
Maternal age	0.022	0.979 (0.94–1.02)	0.345
BMI ≥ 40	0.676	9.748 (2.59–36.66)	0.001
Number of miscarriages	0.166	1.329 (0.96–1.84)	0.087
Number of abortions	0.205	1.394 (0.93–2.08)	0.104
Nicotine abuse	0.462	0.948 (0.38–2.34)	0.907
Prior vaginal bleeding	0.763	0.938 (0.21–4.19)	0.933

## Data Availability

Not applicable.
